# Effects of time pressure and time passage on face-matching accuracy

**DOI:** 10.1098/rsos.170249

**Published:** 2017-06-07

**Authors:** Matthew C. Fysh, Markus Bindemann

**Affiliations:** School of Psychology, University of Kent, Canterbury, UK

**Keywords:** face matching, time pressure, time passage, response bias

## Abstract

This study investigated the impact of time pressure on matching accuracy with face pairs that combined photographs from student ID cards with high-quality person portraits, and under conditions that provided infrequent identity mismatches. Time pressure was administered via two onscreen displays that observers could use to adjust the amount of time that was allocated to a given trial while completing a block of trials within a required timeframe. Under these conditions, observers matched faces under time pressure that varied from 10 to 2 s (Experiment 1) and 8 to 2 s (Experiment 2). An effect of time pressure was found in each experiment, whereby performance deteriorated under time targets of 4 s. Additionally, a match response bias emerged consistently across blocks, and indicated that separately to time pressure, performance also deteriorated due to time passage. These results therefore indicate that both *time passage* and *pressure* exert detrimental effects on face matching.

## 1. Introduction

At border control, passport officers visually compare travellers to their passport photographs. The purpose of this task is to ensure that they are the same person (i.e. an identity match), and not an impostor who is impersonating someone else with a stolen or borrowed passport (i.e. an identity mismatch). This task is known as forensic face matching and is of considerable applied importance. The use of fraudulently obtained genuine passports by such identity impostors is a key enabler of organized crime at the UK border [1], and is a growing security concern [2,3]. However, owing to factors such as inadequately documented arrivals at border control, the true scale of this problem remains unknown [1].

Laboratory studies of face matching provide insight into this problem. When pairs of faces are matched under highly optimized conditions, where stimuli comprise high-quality frontal images of targets taken under even lighting and bearing neutral expressions, observers make up to 20% errors [4,5]. This level of error is already considered problematic for operational contexts, where even small error rates are exacerbated by scale [6,7]. Subsequent research has indicated that factors prevalent in operational contexts reduce accuracy further, such as when to-be-matched stimuli are photographed several months apart [8,9], or when image quality is degraded [10,11]. Other studies have shown also that observers become progressively worse at detecting impostors over a prolonged period [12], even with the administration of regular rest breaks [13].

Further concerns about this task are raised when considering that passport officers have been found to perform at the same level as student observers [14,15]. These findings are corroborated by recent reports that high numbers of fraudulent passport applications go undetected at the issuance stage [16]. Overall, this information suggests that person identification is challenging in applied settings.

In the current study, we investigated a factor that is of practical importance but has only received limited attention in face-matching research, namely the effect of time pressure. Passport officers must often process high volumes of passengers within short timeframes. In the UK, for example, a key performance target for passport officers is to process 95% of passengers from the European Union and European Economic Area within 25 min of joining a passport-control queue on arrival. Similarly, Australian passport officers aim to process 92% of passengers within 30 min. Available information suggests that these passenger processing time targets are frequently missed (e.g. [17–21]). This indicates that passport officers regularly experience high levels of time pressure when processing travellers.

So far, only a few studies have investigated the effect of time pressure on face-matching accuracy. Research currently suggests that under optimized conditions, faces should be viewed for at least 2 s [22,23], but that accuracy can benefit from longer viewing durations under more taxing conditions [24,25]. Taken together, these findings suggest that allowing observers flexibility in the amount of time allocated to each trial, depending on the difficulty of a face-pair stimulus, could reduce errors in this task.

This is an important consideration within the context of passport control, where passport officers can devote more time to processing difficult pairs of faces, provided that this lost time can be either recouped on subsequent trials, or additional time has been accumulated through speeded decisions earlier on. This was recently investigated in one study, where time pressure was administered flexibly using a novel paradigm [26]. In this paradigm, observers used two onscreen displays—a speed gauge and a progress bar—to adjust their response speed to complete each block within a given time target. One important feature of this paradigm is that observers could use these displays to allocate more or less time to a given pair of faces, depending on how far through the block they were, and whether they were on course to meet a time target. The researchers found that under increasing time pressure, face-matching accuracy deteriorated, but improved when time pressure receded, indicating that high time pressure reduces face-matching performance. However, a separate effect of time *passage* was also observed, whereby observers became more likely to erroneously classify face pairs as identity matches as they progressed throughout the task. This match response bias converges with two other studies, where stimuli were also optimized but responses were self-paced [12,13], and suggests that a key factor in face matching is also the passage of time.

These findings raise some important concerns surrounding face matching at passport control, where large numbers of travellers are matched under time pressure that is administered over a sustained duration. However, the effect of time pressure observed by Bindemann *et al*. [26] was numerically small (less than 11%) and it was difficult to specify a consistent time pressure cut-off at which performance deteriorated. Moreover, response times were consistently below 2.5 s, even when up to 10 s were available per trial. These findings might arise as Bindemann *et al*. [26] employed highly optimized stimuli to measure best-possible accuracy under time pressure. As a consequence, however, the extent to which time pressure and the passage of time impact accuracy under more challenging conditions remains unclear. Person identification in relevant applied settings necessitates, for example, the detection of infrequent identity mismatches [4,27], and the matching of a passport bearer with a face photograph that was taken many months or years earlier [9]. The current study explores the effect of time pressure on face-matching accuracy under such conditions.

## 2. Experiment 1

In this experiment, observers matched pairs of faces under time pressure. This was administered via two onscreen displays, which were constantly updated to reflect a person's average response time and the number of trials remaining. These displays were devised as an analogy to passport control at airports, where passport officers are subject to strict passenger processing time targets and can see the number of passengers in a queue that remain to be processed. In the current paradigm, the combined information provided by these displays indicated whether observers were on track to complete a block within a required timeframe. Across five blocks, time pressure systematically increased from 10 to 2 s, or decreased in the reverse order. In addition, this study employed a challenging set of face-pair stimuli, comprising one high-quality image depicting a target under controlled conditions, which was presented alongside a non-controlled student ID photograph that had been taken a minimum of three months earlier. These stimuli were used to more closely explore the impact of time pressure on face-matching accuracy, given that when observers match optimized faces, time pressure exerts only a small numerical effect on performance [26]. To further encapsulate face-matching conditions in practical settings, mismatches occurred infrequently in this task [4,27]. The aim of this design is therefore to indicate how much time observers require to match a challenging set of stimuli, by revealing a cut-off between time pressure and accuracy. Considering that face-matching performance also varies over the duration of the task [12,13,26], the data were also analysed as a function of time passage, by investigating how performance varies over the course of the experiment independently of time pressure.

## 3. Method

### 3.1. Participants

Eighty undergraduates from the University of Kent (17 males and 63 females) with a mean age of 20.5 years (s.d. = 4.4) participated in this study in exchange for course credit or a small fee. Sample size was based on previous studies in this field (e.g. [26]); a *post hoc* analysis also confirmed that this sample size was sufficient to obtain power that satisfies the recommended level of 0.80 [28]. All participants reported normal (or corrected-to-normal) vision.

### 3.2. Stimuli

The stimuli in this study consisted of 200 face pairs from the Kent University Face Database (KUFD), comprising 185 identity matches and 15 mismatches. Each pair comprised a controlled image of a target facing forwards with a neutral expression, which was taken using a 14-megapixel digital camera, against a plain white background under even lighting. These photographs were cropped to depict a target's head and shoulders, and were scaled to a size of 283 × 332 pixels at a resolution of 72 ppi, before being placed on the right-hand side of a plain white canvas. The second image in each pair consisted of a student ID photograph which was retrieved from the University of Kent's online Student Data System, and was taken a minimum of three months before the controlled image. These images were rescaled to a size of 142 × 192 pixels, and were also presented at an image resolution of 72 ppi, before being placed on the left-hand side of the controlled photographs. Mismatching pairs were created by selecting faces that were visually similar regarding hair colour, face and eyebrow shape. These stimuli were divided across five blocks of 40 trials (37 identity matches and three mismatches), with no face appearing more than once.

### 3.3. Time pressure displays

**Figure 1.**
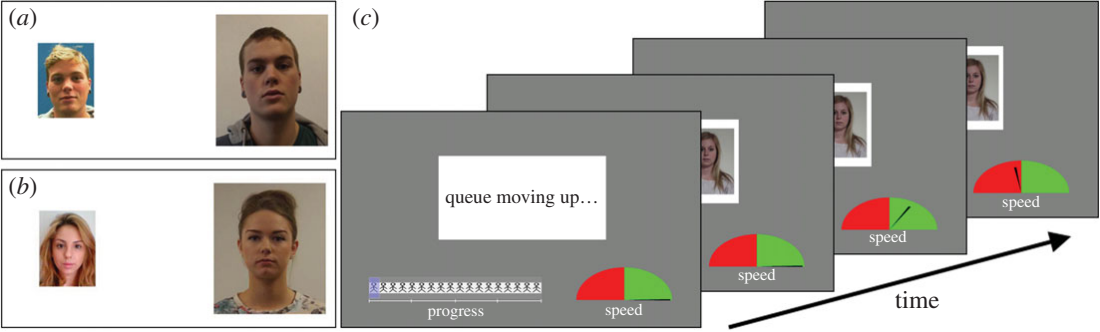
Example identity match (*a*) and mismatch (*b*) pairs used in the study, and an illustration of the stimulus screen and speed displays (*c*).

Time pressure was implemented via two additional onscreen displays, which were presented below the stimuli (for an illustration, see figure 1). One of these displays comprised a queue index indicating the number of trials remaining in the current block. This depicted a row of person icons, to represent a queue of people, and a superimposed progress bar, which advanced on each completed trial. The second display was a semicircular speed gauge which informed participants as to whether they were on track to meet a time target for completing the block. This was evenly divided into a green and a red zone. A dynamic needle was also presented in this display, and reflected whether participants were responding within a given time target (green zone) or were failing to meet this target (red zone). The location of the needle was updated every 100 ms, so that observers could monitor the depletion and accruement of available time in real time. The position of the needle within the speed gauge was based on a person's average response speed, calculated across the number of completed trials in a block, in comparison to the same number of trials multiplied by the set mean time target (i.e. 10, 8, 6, 4 or 2 s), and was proportional to how far participants were behind or ahead of the target time. These displays were reset at the beginning of each block.

### 3.4. Procedure

This experiment was run using PsychoPy software [29]. Each trial was preceded by a 1-s interval screen displaying the message ‘Queue moving up … ’, signalling the onset of the next trial. During this interval, the speed gauge and progress bars remained onscreen, so that observers could monitor their progress and adjust their speed accordingly. This interval screen was replaced with a stimulus display, which remained onscreen until a response was submitted. Participants responded by using one of two keys on a standard computer keyboard, and were instructed to be as accurate as possible at the beginning of the task, as well as between each block.

Participants completed 200 trials, which were counterbalanced across five blocks of 40 face pairs (37 identity matches and three mismatches). At the beginning of the task, participants were instructed that there would be fewer mismatching than matching pairs, but were not informed of the exact ratio. Time pressure was implemented by adjusting the average amount of time that had to be spent on each trial to complete a block within a time target. The order of time pressure was counterbalanced across blocks, such that the available time per trial varied systematically from 10, 8, 6, 4 and 2 s, or vice versa.

These time targets were reflected by the needle within the speed display, which resided in the green zone if an observer was on track to complete a block within the time target, but entered the red zone if a time target was breached. The queue display was updated upon completion of each trial, reflecting how many trials remained in the block. Participants were briefed about these displays at the beginning of the experiment, and were instructed to use these to adjust their response speed accordingly. Specifically, participants were informed that it was acceptable for the needle to enter the red zone if they took more time on some of the trials, provided that lost time could be recouped on later trials. This could be achieved by responding faster on subsequent trials, so that the needle was (back) in the green zone by the end of each block.

## 4. Results

### 4.1. Time pressure

#### 4.1.1. Response times

**Figure 2.**
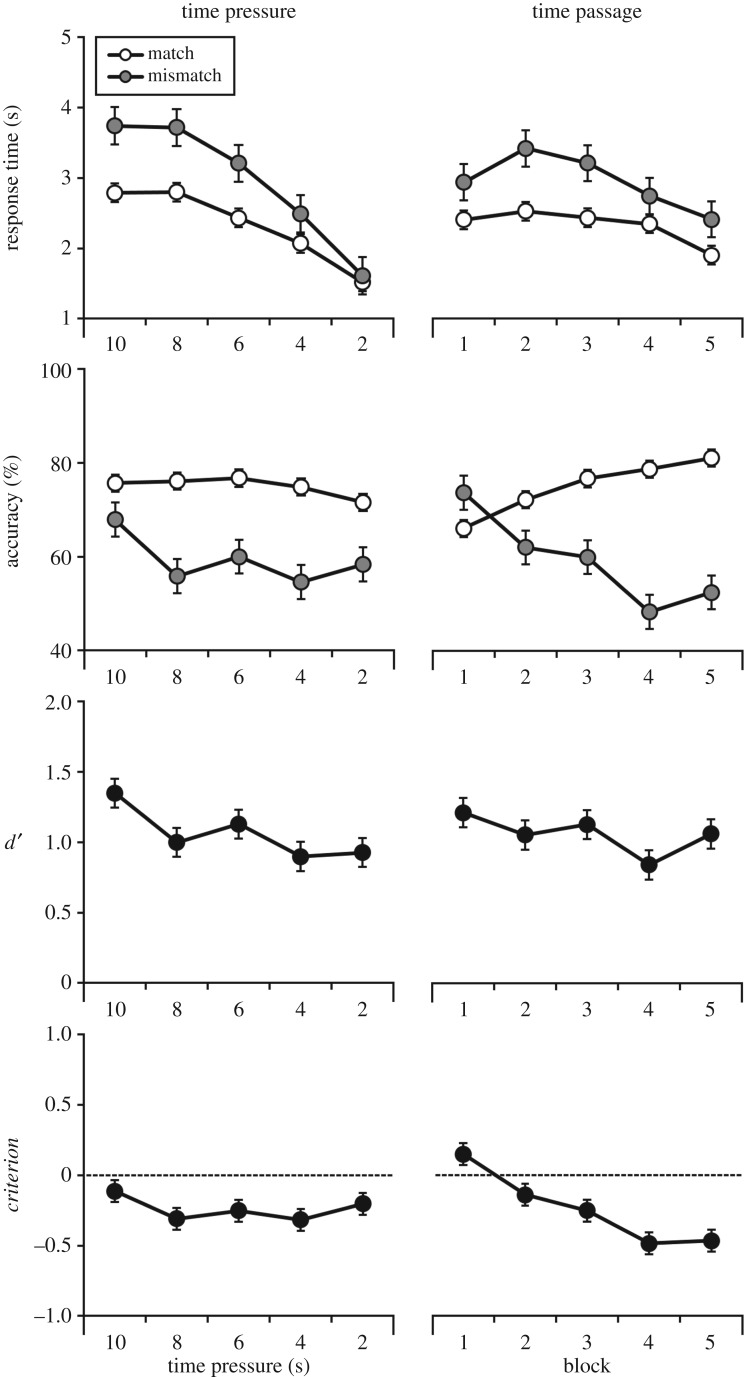
Percentage accuracy, mean correct response times, *d*′, and *criterion* across time pressure conditions, as well as over the passage of time, for Experiment 1. Open circles denote match trials, and grey circles denote mismatch trials. Error bars represent the standard error of the mean.

The response time data were first broken down according to the level of time pressure that was imposed in each block. This showed that all observers complied with the time pressure demands of the task, with the slowest participant taking on average 9.0, 7.3, 5.6, 3.5 and 2.0 s in Blocks 1–5, respectively. Next, the data were broken down further into mean correct response times on match and mismatch trials, which are depicted in figure 2, and were analysed using a 2 (trial: match versus mismatch) × 5 (time pressure: 10, 8, 6, 4, 2 s) within-subjects analysis of variance (ANOVA). This revealed an effect of trial, *F*_1,48_ = 12.62, *p* < 0.01, $\eta_{p}^{2}=0.21,$ which was due to faster responses on match trials. In addition, there was an effect of time pressure, *F*_4,192_ = 20.50, *p* < 0.001, $\eta_{p}^{2}=0.30.$ Bonferroni-adjusted pairwise comparisons showed that responses were fastest in the 2-s block, all *p*s < 0.001, followed by the 4-s block, all *p*s < 0.05, but were comparable between the 6-, 8- and 10-s blocks, all *p*s ≥ 0.27. The interaction between time pressure and trial was not significant, *F*_4,192_ = 1.33, *p* = 0.26, $\eta_{p}^{2}=0.03.$

#### 4.1.2. Accuracy

Next, the percentage accuracy data for each time pressure condition were calculated. These scores are also depicted in figure 2, and reflect that under 2 s of time pressure, accuracy on mismatch trials deteriorated to 58%, while performance on match trials appeared comparable across all time pressure conditions. A 2 (trial) × 5 (time pressure) within-subjects ANOVA found an effect of trial, due to higher accuracy on match trials, *F*_1,79_ = 20.12, *p* < 0.001, $\eta_{p}^{2}=0.20,$ as well as an effect of time pressure, *F*_4,316_ = 3.91, *p* < 0.01, $\eta_{p}^{2}=0.05.$ Bonferroni-adjusted comparisons showed that this was due to higher accuracy when time pressure was 10 s, compared to 4 and 2 s, both *p*s < 0.05. The difference in accuracy between 10 and 8 s was also approaching significance, *p* = 0.05. However, no other comparisons were significant, all *p*s ≥ 0.60, and these factors did not interact, *F*_4,316_ = 2.26, *p* = 0.06, $\eta_{p}^{2}=0.03$.

#### 4.1.3. *d*′ and *criterion*

For completeness, the percentage accuracy data were also converted to signal detection measures *d*′ and *criterion*, to measure overall sensitivity (accuracy) and response bias, respectively. For *d*′, a one-way within-subjects ANOVA revealed a small but significant effect of time pressure, *F*_4,316_ = 3.66, *p* < 0.01, $\eta_{p}^{2}=0.04,$ due to lower sensitivity under 4 and 2 s of time pressure compared with the 10-s condition, both *p*s < 0.05. However, sensitivity was comparable between all other blocks, all *p*s ≥ 0.09.

The analogous analysis of *criterion* did not find an effect of time pressure, *F*_4,316_ = 2.06, *p* = 0.09, $\eta_{p}^{2}=0.03,$ indicating that observers' response patterns did not vary across time pressure conditions. However, this does not rule out the possibility that a response bias was present throughout the task. To explore this further, therefore, *criterion* in each block was compared to zero using a series of one-sample *t*-tests. This revealed that response criterion was close to zero under 10 s of time pressure, *t*_79_ = 1.48, *p* = 0.14, but was reliably below zero when time pressure was 8 s, *t*_79_ = 4.07, *p* < 0.001, 6 s, *t*_79_ = 3.42, *p* < 0.01, 4 s, *t*_79_ = 4.13, *p* < 0.001 and 2 s, *t*_79_ = 2.46, *p* < 0.05. These results indicate that a match response bias was present in each time pressure condition except the 10-s block.

### 4.2. Time passage

#### 4.2.1. Response times

Next, the data were analysed according to time *passage*. For this purpose, the data were collapsed across increasing and decreasing time pressure conditions and analysed by block order. As with the analysis of time pressure, mean correct response times were analysed first, and are displayed in figure 2. A 2 (trial: match versus mismatch) × 5 (block: 1, 2, 3, 4, 5) within-subjects ANOVA revealed an effect of trial, *F*_1,48_ = 12.62, *p* < 0.01, $\eta_{p}^{2}=0.21,$ due to faster responses on match trials. In addition, an effect of block was found, *F*_4,192_ = 4.50, *p* < 0.01, $\eta_{p}^{2}=0.09,$ due to faster responses in Block 5 compared to Blocks 2, 3 and 4, all *p*s < 0.05. However, no further comparisons were significant, all *p*s ≥ 0.20, and trial type did not interact with block, *F*_4,192_ = 0.99, *p* = 0.42, $\eta_{p}^{2}=0.02$.

#### 4.2.2. Accuracy

Percentage accuracy scores were calculated for each block, collapsed across order of time pressure. These data are also depicted in figure 2, and reflect that performance on mismatch trials deteriorated across blocks, from 74% in Block 1 to 53% in Block 5. A 2 (trial) × 5 (block) within-subjects ANOVA revealed an effect of trial, *F*_1,79_ = 20.10, *p* < 0.001, $\eta_{p}^{2}=0.20,$ as well as of block, *F*_4,316_ = 2.46, *p* < 0.05, $\eta_{p}^{2}=0.03,$ and a significant interaction, *F*_4,316_ = 24.59, *p* < 0.001, $\eta_{p}^{2}=0.24$.

Simple main effects analysis for this interaction revealed that accuracy on match trials improved across blocks, *F*_4,76_ = 29.67, *p* < 0.001, $\eta_{p}^{2}=0.61,$ with Bonferroni-adjusted pairwise comparisons showing that accuracy was higher in all blocks following the first and second block, all *p*s < 0.01, as well as in the final block compared to Block 3, *p* < 0.01. However, performance was comparable between Blocks 4 and 5, and between Blocks 3 and 4, both *p*s ≥ 0.19. The deterioration on mismatch trials was also significant, *F*_4,76_ = 8.66, *p* < 0.001, $\eta_{p}^{2}=0.31,$ with worse accuracy in all blocks following Block 1, all *p*s < 0.05, as well as in Block 4 compared to Block 2, *p* < 0.01. Performance was comparable between the remaining blocks, all *p*s ≥ 0.10.

A simple main effect of trial was also found in the second, *F*_1,79_ = 5.73, *p* < 0.05, $\eta_{p}^{2}=0.07,$ third, *F*_1,79_ = 13.52, *p* < 0.001, $\eta_{p}^{2}=0.15,$ fourth, *F*_1,79_ = 41.57, *p* < 0.001, $\eta_{p}^{2}=0.35,$ and final blocks, *F*_1,79_ = 33.80, *p* < 0.001, $\eta_{p}^{2}=0.30,$ reflecting higher accuracy on match compared to mismatch trials. By contrast, mismatch accuracy was higher than match accuracy in Block 1, but this difference failed to reach significance, *F*_1,79_ = 3.91, *p* = 0.05, $\eta_{p}^{2}=0.05$.

#### 4.2.3. *d*′ and *criterion*

Percentage accuracy scores were again transformed into *d*′ and *criterion*. The analysis of *d*′ revealed that overall sensitivity was comparable across blocks, *F*_4,316_ = 2.00, *p* = 0.10, $\eta_{p}^{2}=0.03$. However, there was an effect of block on *criterion* scores, *F*_4,316_ = 26.28, *p* < 0.001, $\eta_{p}^{2}=0.25,$ due to a significantly lower response criterion in Blocks 4 and 5 compared to all preceding blocks, all *p*s < 0.05, as well as in Blocks 2 and 3 compared to the first block, both *p*s < 0.001. However, *criterion* was comparable between the second and third block, *p* = 0.84.

As before, these scores were also compared to zero. One-sample *t*-tests revealed that *criterion* was above zero in Block 1, *t*_79_ = 2.42, *p* < 0.05, due to a higher number of mismatch responses at the beginning of the task. By contrast, *criterion* was below zero in the second, *t*_79_ = 2.06, *p* < 0.05, third, *t*_79_ = 3.42, *p* < 0.01, fourth, *t*_79_ = 6.14, *p* < 0.001, and final block, *t*_79_ = 5.60, *p* < 0.001. These results indicate that over time, observers became increasingly likely to classify faces as identity matches.

## 5. Discussion

This experiment investigated the effects of time pressure and time passage on face-matching performance. Time pressure appeared to specifically impact performance on mismatch trials, whereby accuracy deteriorated as the average time target per trial was reduced. This is evident from *d*′, which reflected that performance was worst in the 4- and 2-s conditions. Response criterion did not vary across the different levels of time pressure, but was reliably below zero in all conditions following the 10-s condition, reflecting that observers were more prone to classifying stimuli as identity matches in the 8-, 6-, 4- and 2-s conditions. This bias could be attributed to observers' knowledge that mismatches would be occurring less frequently than matches over the task. However, other research has shown that even when match and mismatch trials occur with equal frequency, a similar response bias emerges, but is exacerbated by time pressure [26].

In addition, an effect of time passage was also observed in this experiment, where accuracy on match trials improved from 66 to 81% between Blocks 1 and 5, but also deteriorated on mismatch trials from 74 to 53%. This pattern reflects a shift in response criterion and shows that observers adopted a bias to classify more face pairs as identity matches over time.

These findings are consistent with those of Bindemann *et al*. [26], where face matching was most error-prone under 2 s of time pressure. Likewise, performance in the current experiment was lowest under 4- and 2-s time targets, but did not differ between these conditions. In addition, these findings converge with studies that found a response bias to emerge over time, whereby observers make an increasing number of erroneous identity match responses over the course of an experiment [12,13].

## 6. Experiment 2

Experiment 1 indicates that time pressure and time passage exert distinct effects on face-matching performance. Time pressure reduces accuracy (%) and sensitivity (*d*′) as the time available to match faces decreases, but does not affect observers' decision *criterion*. By contrast, sensitivity (*d*′) is not affected by time passage, but *criterion* decreases over the course of the experiment, reflecting a bias to make increasingly more identity-match decisions. However, not all aspects of the results were clear-cut. For example, the time pressure analysis also revealed a match response bias (*criterion*) in all conditions except for the 10-s condition, and a marginally non-significant interaction of time pressure and trial type.

The aim of Experiment 2 was therefore twofold. Firstly, we sought to replicate the distinct effects that time pressure and time passage appear to exert in Experiment 1. Secondly, we sought to clarify marginal effects, such as the non-significant interaction of time pressure and trial type. Considering that observers' mean response times were substantially below the target time of the 10-s condition in Experiment 1, we excluded this condition in Experiment 2. In turn, this exclusion enabled us to increase the number of data points for each time pressure condition, by distributing surplus trials across the remaining blocks. Thus, in Experiment 2 observers completed four blocks of face-matching trials, where time pressure varied among 8, 6, 4 and 2 s.

## 7. Method

### 7.1. Participants

Sixty undergraduates (10 males and 50 females) with a mean age of 20 years (s.d. = 3.3) participated in this experiment in exchange for course credit or a small fee. None of these had participated in the previous experiment, and all reported normal, or corrected-to-normal vision.

### 7.2. Stimuli and procedure

As in the previous experiment, this experiment featured 200 face pairs extracted from the KUFD. One identity match from Experiment 1 was replaced with a mismatch trial, resulting in 184 match trials and 16 mismatches. These were evenly divided over four blocks of 50 face pairs (46 match and 4 mismatch), and were counterbalanced across participants, with no pair appearing more than once for each observer.

The procedure was identical to the previous experiment, except for the difference that instead of five blocks, where time pressure increased or decreased from 10 to 2 s, this task comprised four blocks, with time targets varying systematically from 8 to 2 s. To further encapsulate time pressure, the interval between each trial was reduced to 500 ms, and observers could receive up to three verbal prompts per block. These consisted of ‘please speed up’, ‘you must speed up’ and ‘go faster!’, and were only issued if the needle was in the red zone at 25%, 50% and 75% block completion, respectively. All other aspects of the procedure, such as the speed gauge and the progress bar, were unchanged.

## 8. Results

### 8.1. Time pressure

#### 8.1.1. Response times

**Figure 3.**
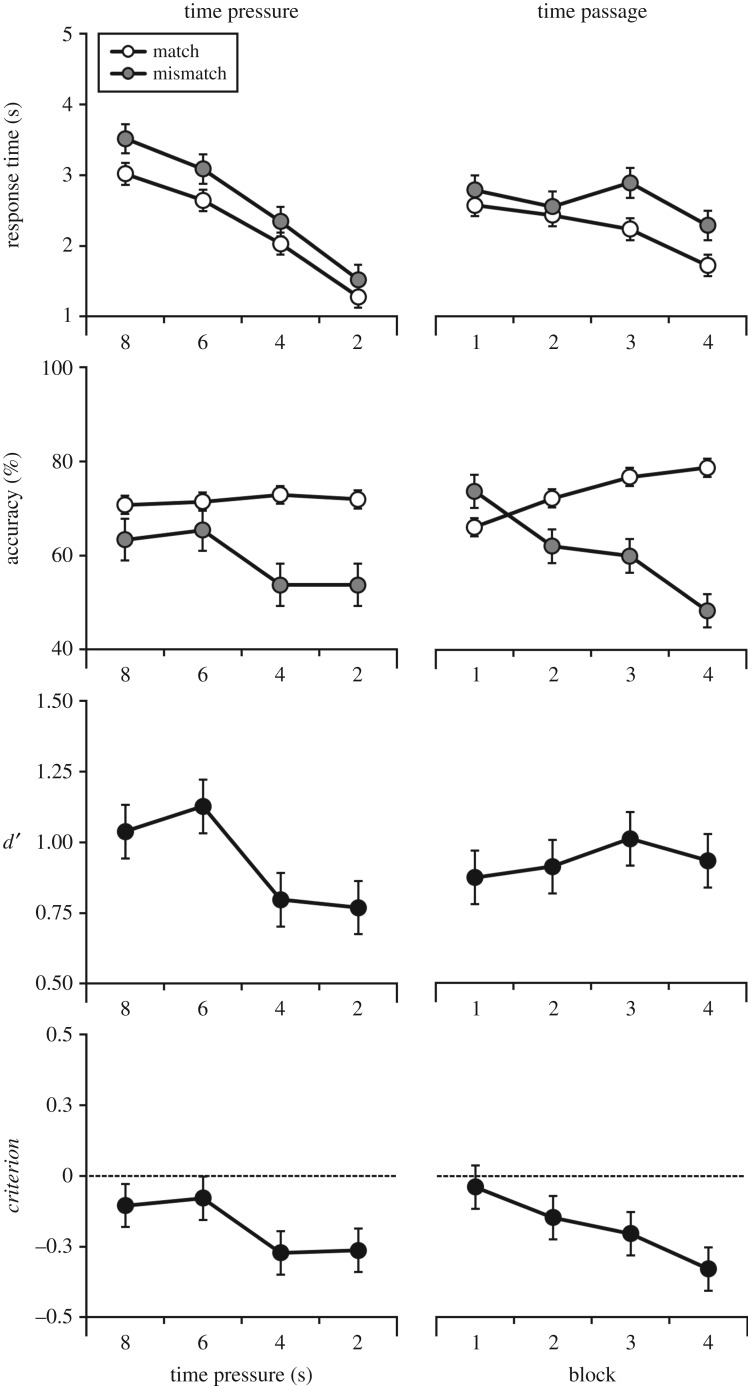
Percentage accuracy, mean correct response times, *d*′, and *criterion* across time pressure conditions, as well as over the passage of time, for Experiment 2. Open circles denote match trials, and grey circles denote mismatch trials. Error bars represent the standard error of the mean.

As in Experiment 1, response times were analysed first. The slowest observer took 7.3, 5.1, 3.3 and 1.4 s to complete the 8-, 6-, 4- and 2-s conditions, respectively. These data were next broken down into mean correct response times on match and mismatch trials, which are depicted in figure 3. A 2 (trial: match versus mismatch) × 4 (time pressure: 8, 6, 4, 2 s) within-subjects ANOVA revealed that responses on match trials were faster than on mismatch trials, *F*_1,52_ = 17.00, *p* < 0.001, $\eta_{p}^{2}=0.25$. There was also an effect of time pressure, *F*_3,156_ = 50.75, *p* < 0.001, $\eta_{p}^{2}=0.49,$ due to faster responses in the 2-s condition compared to the 4-, 6- and 8-s conditions, all *p*s < 0.001. In addition, responses in the 4-s condition were faster than in the 6- and 8-s conditions, both *p*s < 0.001. The difference between the 6- and 8-s conditions was approaching significance, *p* = 0.05. These factors did not interact, *F*_3,156_ = 0.82, *p* = 0.48, $\eta_{p}^{2}=0.02$.

#### 8.1.2. Accuracy

Percentage accuracy scores for this experiment are also depicted in figure 3, and show that under 4 and 2 s of time pressure, accuracy on mismatch trials deteriorated to 54%, while performance on match trials remained comparable across all time pressure conditions. To analyse these data, a 2 (trial: match versus mismatch) × 4 (time pressure: 8, 6, 4 and 2 s) within-subjects ANOVA was conducted. This revealed an effect of time pressure, *F*_3,177_ = 3.71, *p* < 0.05, $\eta_{p}^{2}=0.06,$ as well as an effect of trial, *F*_1,59_ = 10.58, *p* < 0.01, $\eta_{p}^{2}=0.15,$ and an interaction, *F*_3,177_ = 4.72, *p* < 0.01, $\eta_{p}^{2}=0.07$.

Simple main effects analysis revealed that performance on match and mismatch trials was comparable in the 6-s, *F*_1,59_ = 1.64, *p* = 0.21, $\eta_{p}^{2}=0.03,$ and 8-s conditions, *F*_1,59_ = 2.23, *p* = 0.14, $\eta_{p}^{2}=0.04,$ whereas accuracy was higher on match trials in the 4-s, *F*_1,59_ = 15.90, *p* < 0.001, $\eta_{p}^{2}=0.21$, and 2-s conditions, *F*_1,59_ = 15.36, *p* < 0.001, $\eta_{p}^{2}=0.21$. In addition, there was a simple main effect of time pressure on mismatch trials, *F*_3,57_ = 5.07, *p* < 0.01, $\eta_{p}^{2}=0.21$. Bonferroni-adjusted pairwise comparisons showed that this was due to higher accuracy in the 6-s condition compared with the 4-s condition, *p* < 0.05. However, performance was comparable between all other conditions, all *p*s ≥ 0.07. There was no effect of time pressure on match trials, *F*_3,57_ = 0.68, *p* = 0.57, $\eta_{p}^{2}=0.03$.

#### 8.1.3. *d*′ and *criterion*

The percentage accuracy data were converted to signal detection measures *d*′ and *criterion* to measure overall performance and response bias. For *d*′, ANOVA found an effect of time pressure, *F*_3,177_ = 4.00, *p* < 0.01, $\eta_{p}^{2}=0.06,$ due to worse performance under 4 s of time pressure compared with 6 s, *p* < 0.05. However, sensitivity was comparable across all other blocks, all *p*s ≥ 0.07. The analogous analysis of *criterion* also revealed an effect of time pressure, *F*_3,177_ = 4.04, *p* < 0.01, $\eta_{p}^{2}=0.06,$ which was due to a shift in response criterion between the 4- and 6-s block, *p* < 0.05. No other comparisons reached significance, all *p*s ≥ 0.11.

In an additional step, the *criterion* scores for each time pressure condition were also compared to zero using one-sample *t*-tests. This revealed that *criterion* was comparable to zero under 8 s, *t*_59_ = 1.31, *p* = 0.20, and 6 s of time pressure, *t*_59_ = 1.05, *p* = 0.30, but was reliably below zero under 4 s, *t*_59_ = 3.63, *p* < 0.01, and 2 s of time pressure, *t*_59_ = 3.58, *p* < 0.01. This shows that under strict time pressure targets of 4 and 2 s, observers exhibit a bias to classify more face pairs as depicting the same person.

### 8.2 Time passage

#### 8.2.1 Response times

Next, the data were analysed according to time *passage*. These data are displayed in figure 3, and reflect that responses generally became faster over time. A 2 (trial: match versus mismatch) × 4 (block: 1, 2, 3, 4) within-subjects ANOVA revealed an effect of trial, *F*_1,52_ = 17.00, *p* < 0.001, $\eta_{p}^{2}=0.25,$ due to faster responses on identity match trials. There was also an effect of block, *F*_3,156_ = 2.99, *p* < 0.05, $\eta_{p}^{2}=0.05,$ due to faster responses in the final block compared to the third, *p* < 0.01. However, no further comparisons were significant, all *p*s ≥ 0.28, and these factors did not interact, *F*_3,156_ = 2.40, *p* = 0.07, $\eta_{p}^{2}=0.04$.

#### 8.2.2. Accuracy

To determine whether performance was declining over time, we next examined percentage accuracy scores for each block. Breaking down the data in this way revealed that accuracy on mismatch trials decreased from 63% in Block 1, to 54% in Block 4. Conversely, performance on identity-match trials improved over time, from 67% in Block 1 to 76% in Block 4. A 2 (trial) × 4 (block) within-subjects ANOVA did not reveal an effect of block, *F*_3,177_ = 0.23, *p* = 0.88, $\eta_{p}^{2}=0.00,$ but an effect of trial, *F*_1,59_ = 10.58, *p* < 0.01, $\eta_{p}^{2}=0.15,$ and a significant interaction, *F*_3,177_ = 5.24, *p* < 0.01, $\eta_{p}^{2}=0.08$.

Simple main effects analysis for this interaction revealed that performance on match and mismatch trials was comparable in Block 1, *F*_1,59_ = 0.88, *p* = 0.35, $\eta_{p}^{2}=0.02,$ but was significantly higher on match trials in the second, *F*_1,59_ = 5.28, *p* < 0.05, $\eta_{p}^{2}=0.08,$ third, *F*_1,59_ = 8.42, *p* < 0.01, $\eta_{p}^{2}=0.13,$ and fourth block, *F*_1,59_ = 18.21, *p* < 0.001, $\eta_{p}^{2}=0.24$. In addition, a simple main effect of block was found on match trials, *F*_3,57_ = 9.75, *p* < 0.001, $\eta_{p}^{2}=0.34$. Bonferroni-adjusted comparisons showed that accuracy was higher in Block 4 compared to Blocks 1 and 2, both *p*s < 0.001, and in Block 3 compared to Block 1, *p* < 0.001. However, performance was comparable between the second and third, and the second and first block, both *p*s ≥ 0.09. The deterioration on mismatch trials was not significant, *F*_3,57_ = 1.70, *p* = 0.18, $\eta_{p}^{2}=0.08$.

#### 8.2.3. *d*′ and *criterion*

The percentage accuracy data were again converted into *d*′ and *criterion*. For *d*′, ANOVA did not reveal an effect of block, *F*_3,177_ = 0.41, *p* = 0.75, $\eta_{p}^{2}=0.01$. However, this effect was present for *criterion*, *F*_3,177_ = 5.81, *p* < 0.01, $\eta_{p}^{2}=0.09,$ which was lower in the final block, compared with Block 1, *p* < 0.01. No other comparisons were significant, all *p*s ≥ 0.10. One-sample *t*-tests were conducted to compare the *criterion* scores in each block to zero. This analysis revealed that *criterion* was comparable to zero in Block 1, *t*_59_ = 0.58, *p* = 0.56, but was reliably below zero in the second, *t*_59_ = 2.06, *p* < 0.05, third, *t*_59_ = 2.53, *p* < 0.05, and final block, *t*_59_ = 4.00, *p* < 0.001. This indicates that a bias emerged after Block 1 to classify face pairs as identity matches.

## 9. Discussion

This experiment found that face-matching performance again deteriorated under time pressure targets of 4 and 2 s. Numerically, this effect accounted for 11% of errors on mismatch trials between the 8- and 4-s conditions and provides evidence that time pressure is detrimental to the detection of mismatching identities. Moreover, a reduction in *d*′ was observed between the 6- and 4-s conditions, in conjunction with a match response bias. Overall, these findings suggest that 4 s represents a possible mean cut-off time at which face matching deteriorates, due to a bias to classify face pairs as identity matches.

Converging with the previous experiment, the separate analysis of time passage also revealed this match response bias across blocks. Owing to this bias, performance on match trials improved from 67 to 76% in Blocks 1–4. This provides further evidence that over the passage of time, observers become more prone to perceive two faces in a pair as the same identity.

## 10. General discussion

This study investigated the effects of time pressure on face-matching accuracy. Across two experiments, time pressure was administered flexibly via two onscreen displays that allowed observers to monitor whether they were on track to meet a time target, or were required to speed up (see [26] for a similar design). The effect of time pressure was clearest in response times, which decreased gradually across the 6, 4 and 2 s conditions in both experiments. Importantly, both experiments also revealed an effect of time pressure on accuracy, where *d*′ deteriorated at 4 s relative to more liberal time targets, but was comparable to time targets of 2 s. However, in numerical terms, these effects were relatively small, accounting for only 7 and 5% additional errors in the 4-s compared to the 10- and 6-s conditions in Experiment 1 and 2, respectively.

These findings converge with recent work where time pressure exerted only a small effect on face-matching performance, and accounted for less than 11% of errors [26]. We reasoned *a priori* that this small effect might have been due to the optimized stimuli employed in this research, for which accuracy is generally high (e.g. [5,30]). Contrary to this prediction, however, the current study obtained a comparable effect of time pressure on face-matching performance. This occurred in a context where general performance was considerably poorer than that observed by Bindemann *et al*. [26]. This poor general performance converges with additional work where to-be-compared stimuli portrayed more within-person variation [9], and mismatches were rare [27]. Taken together, these findings suggest that time pressure only exerts a relatively moderate effect on face matching, both with optimized stimuli [26] and under the more taxing conditions of the current experiments.

It is worth noting that these results were obtained in a context where average response times were consistently below the target threshold in all time pressure conditions. In the 8-s condition, for example, average response times of 3.0 and 3.2 s were obtained for Experiment 1 and 2, respectively. These fast responses are surprising given that observers were instructed at the beginning to use the onscreen displays to adjust their speed accordingly. Similar response patterns were observed by Bindemann *et al*. [26], who found that response times were consistently below 2.5 s even when 10 s were available per trial. The researchers considered whether this could be due to a lack of motivation from student observers to fully use the available time on each trial. An alternative explanation could be that observers consistently underestimated the difficulty of matching unfamiliar faces. This makes sense when considering studies where observers generalize their ability to match and identify familiar faces, which is comparatively high, to the more difficult identification of unfamiliar faces, and so fail to anticipate errors that arise in such tasks [31,32]. This is also supported by evidence that passport officers take longer than students in face-matching tasks but are not more accurate [14]. At present, however, it is unclear how observers allocate their processing time on a trial-by-trial basis in face matching. Research suggests that some expert observers incur greater benefits than student controls when additional time is provided [25]. This indicates that there is an effective strategy of time allocation in face matching, and should be explored in future research.

Although only a small number of errors could be attributed to time pressure in the current study, a strong effect of time *passage* was also consistently detected in both experiments. This was characterized by a match response bias that emerged over time, and accounted for up to 21% of errors on mismatch trials. In numerical terms, the passage of time therefore appears to exert a more detrimental effect on face-matching accuracy than time pressure, particularly on the detection of identity mismatches. This time passage effect has also been demonstrated in three other studies, where mismatch accuracy deteriorated to below chance levels when optimized faces were matched under self-paced conditions [12,13] and under time pressure [26]. This therefore appears to be a robust effect, although its cause remains unclear [13].

In this study, it is notable that the effects of time pressure and time passage were obtained through separate analysis, for which the data were ordered either by the time pressure conditions, which were counterbalanced across observers, or by block order in the experiment. These data transformations as well as the different characteristics of time pressure and time passage demonstrate that these effects are qualitatively different, but can concurrently influence face matching. This raises concerns for applied settings that rely on face matching, such as person identification at passport control. In those settings, personnel experience time pressure frequently (e.g. [18–21]) while also performing face matching over prolonged periods. Time pressure effects may be exacerbated further in applied settings by the requirement to check additional person information, such as names, nationality and travel documents [33,34]. The current experiments, which encompassed only 200 trials per participant and required face matching only, may therefore still underestimate the impact of time pressure and time passage in applied settings [12,13].

## Supplementary Material

Supplementary analysis for Experiments 1 and 2

## Supplementary Material

Raw data

## Data accessibility

The data for this research are openly available for download from Dryad Digital Repository: http://dx.doi.org/10.5061/dryad.vm0m6 [35].
