# Correction to ‘Effects of time pressure and time passage on face-matching accuracy’

**DOI:** 10.1098/rsos.171159

**Published:** 2017-09-13

**Authors:** Matthew C. Fysh, Markus Bindemann

*R. Soc. open sci.* **4**, 170249. (Published 7 June 2017). (doi:10.1098/rsos.170249)

This article contains an error in figure 3, whereby the percentage accuracy data depicted for *time passage* were calculated from scores in Experiment 1, as opposed to Experiment 2. All other graphs in this figure portray the correct values. The amended figure is depicted below. We regret that this mistake occurred in the first place; however, add that this does not change the scientific findings of the paper. We apologize for any confusion that this may have caused.

**Figure 3.**
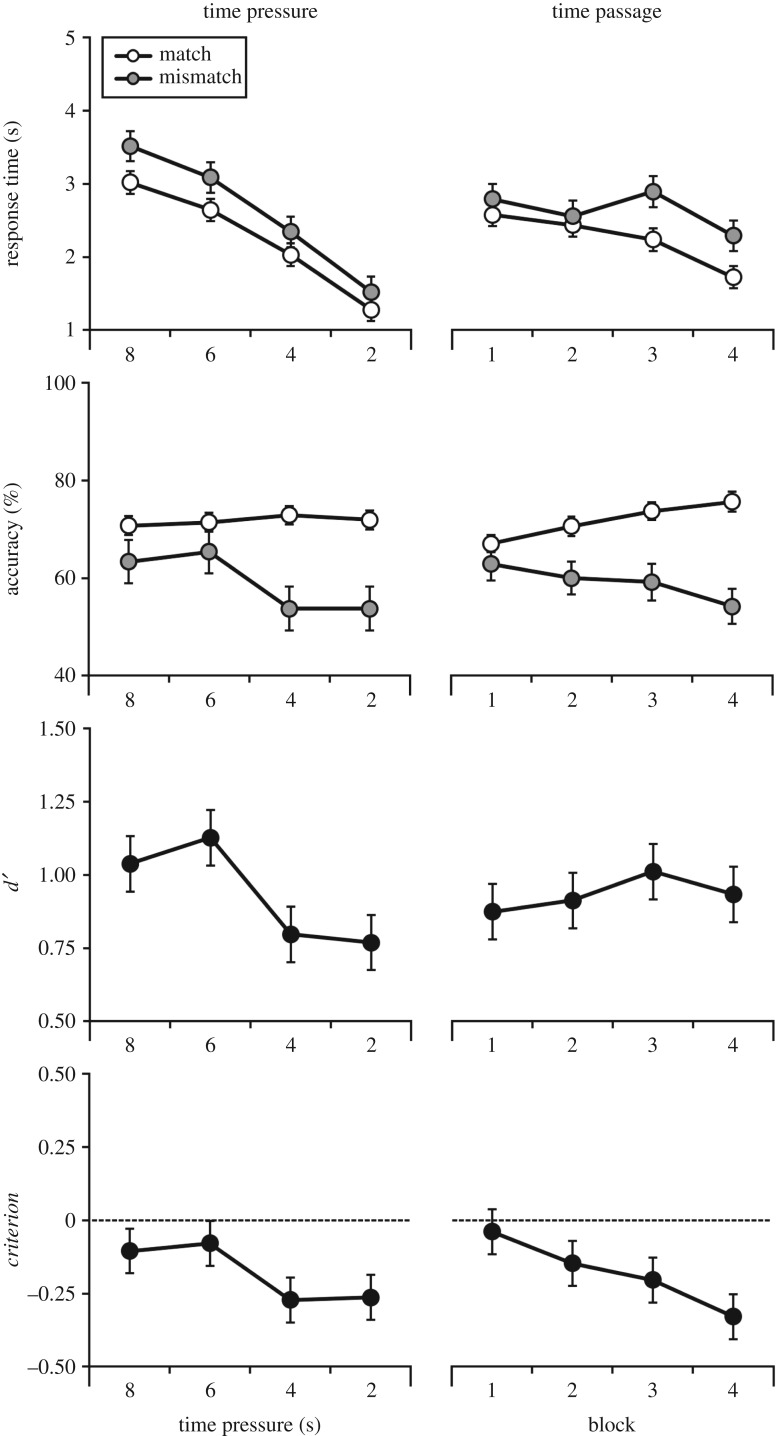
Percentage accuracy, mean correct response times, *d*′, and *criterion* across time pressure conditions, as well as over the passage of time, for Experiment 2. Open markers denote match trials, and grey markers denote mismatch trials. Error bars represent the standard error of the mean.

