# Supplementary material for: Effects of time pressure and time passage on face-matching accuracy
Source: R Soc Open Sci. 2017 Jun 7;4(6):170249. doi: 10.1098/rsos.170249 (PMC5493919; doi:10.1098/rsos.170249)
Supplement: Supplementary analysis for Experiments 1 and 2 [file rsos170249supp1.doc]

**APPENDIX**

**Supplementary Data - Experiment 1**

*Response times*

A 2 (time pressure: increasing vs. decreasing) x 2 (trial: match vs. mismatch) x 5 (time: 10, 8, 6, 4, 2 seconds) mixed-factor ANOVA revealed an effect of time pressure, *F*(1,47) = 12.03, *p* < 0.01, η_p_^2^ = 0.20, and of trial, *F*(1,47) = 13.75, *p* < 0.01, η_p_^2^ = 0.23, as well as an interaction between these factors, *F*(1,47) = 5.80, *p* < 0.05, η_p_^2^ = 0.11. In addition, an effect of time was found, *F*(4,188) = 5.95, *p* < 0.001, η_p_^2^ = 0.11, which also interacted with time pressure condition, *F*(4,188) = 20.57, *p* < 0.001, η_p_^2^ = 0.30. However, there was no interaction between trial and time, *F*(4,188) = 0.96, *p* = 0.43, η_p_^2^ = 0.02, and the three-way interaction was not significant, *F*(4,188) = 1.51, *p* = 0.20, η_p_^2^ = 0.03.

For the interaction between time pressure and trial, simple main effects analysis revealed that under decreasing time pressure, responses were faster on match, *F*(1,47) = 9.59, *p* < 0.01, η_p_^2^ = 0.17, and mismatch trials, *F*(1,47) = 11.91, *p* < 0.01, η_p_^2^ = 0.20, compared to when time pressure was increasing. A simple main effect of trial type was also found in the increasing time pressure condition, *F*(1,47) = 19.09, *p* < 0.001, η_p_^2^ = 0.29, due to faster responses on match trials. However, response times were comparable between match and mismatch trials when time pressure was decreasing, *F*(1,47) = 0.83, *p* = 0.37, η_p_^2^ = 0.02.

Analysis of the interaction between time pressure condition and time found that responses remained comparable across blocks when time pressure was decreasing, *F*(4,44) = 1.33, *p* = 0.27, η_p_^2^ = 0.11. However, there was a simple main effect of time under increasing time pressure, *F*(4,44) = 12.52, *p* < 0.001, η_p_^2^ = 0.53. Bonferroni-adjusted pairwise comparisons revealed that responses in the 2-second block were faster than in all other blocks, all *p*s < 0.001. In addition, responses in the 4-second block were faster than in the 6-, 8-, and 10-second blocks, all *p*s < 0.01. No other comparisons reached significance, all *p*s ≥ 0.12. Simple main effects analysis also revealed that responses were faster in Block 1 of the decreasing time pressure condition (2 seconds) compared to Block 1 of the increasing time pressure condition (10 seconds), *F*(1,47) = 23.50, *p* < 0.001, η_p_^2^ = 0.33. Moreover, responses were faster in Block 2 (4 seconds), *F*(1,47) = 22.38, *p* < 0.001, η_p_^2^ = 0.32, and Block 3 (6 seconds), *F*(1,47) = 12.66, *p* < 0.01, η_p_^2^ = 0.21, of the decreasing time pressure condition, compared to Block 2 (8 seconds) and Block 3 (6 seconds) under increasing time pressure. Responses were comparable between conditions in Block 4, *F*(1,47) = 0.89, *p* = 0.35, η_p_^2^ = 0.02, but were faster in Block 5 of the increasing time pressure condition (2 seconds), compared to the decreasing time pressure condition (10 seconds), *F*(1,47) = 5.16, *p* < 0.05, η_p_^2^ = 0.10.

*Accuracy*

A 2 (time pressure) x 2 (trial) x 5 (time) mixed-factor ANOVA did not reveal an effect of time pressure condition, *F*(1,78) = 0.53, *p* = 0.47, η_p_^2^ = 0.01, but of trial, *F*(1,78) = 19.91, *p* < 0.001, η_p_^2^ = 0.20, and time, *F*(4,312) = 2.52, *p* < 0.05, η_p_^2^ = 0.03. There was also an interaction between trial and time, *F*(4,312) = 24.32, *p* < 0.001, η_p_^2^ = 0.24, and between time pressure and time, *F*(4,312) = 2.87, *p* < 0.05, η_p_^2^ = 0.04. Time pressure did not interact with trial, *F*(1,78) = 1.12, *p* = 0.30, η_p_^2^ = 0.01, and the three-way interaction was not significant, *F*(4,312) = 0.45, *p* = 0.78, η_p_^2^ = 0.01.

For the interaction between time pressure condition and time, simple main effects analysis revealed that accuracy was worse in Block 1 when time pressure was decreasing (2 seconds) than when increasing (10 seconds), *F*(1,78) = 8.16, *p* < 0.01, η_p_^2^ = 0.10, but was comparable between time pressure conditions in the second, *F*(1,78) = 0.99, *p* = 0.32, η_p_^2^ = 0.01, third, *F*(1,78) = 0.07, *p* = 0.80, η_p_^2^ = 0.001, fourth, *F*(1,78) = 0.01, *p* = 0.92, η_p_^2^ = 0.00, and fifth block, *F*(1,78) = 1.99, *p* = 0.16, η_p_^2^ = 0.03. A simple main effect of time was also found in the increasing time pressure condition, *F*(4,75) = 3.36, *p* < 0.01, η_p_^2^ = 0.15. Bonferroni-adjusted pairwise comparisons revealed that accuracy was worse in Block 4 (4 seconds) and Block 5 (2 seconds) compared to Block 1 (10 seconds), both *p*s < 0.05. However, no other comparisons were significant, all *p*s ≥ 0.17, and accuracy was comparable between blocks in the decreasing time pressure condition, *F*(4,75) = 1.06, *p* = 0.38, η_p_^2^ = 0.05.

For the interaction between trial and time, accuracy on match trials was superior to mismatch trials in the second, *F*(1,78) = 5.64, *p* < 0.05, η_p_^2^ = 0.07, third, *F*(1,78) = 13.22, *p* < 0.001, η_p_^2^ = 0.15, fourth, *F*(1,78) = 40.90, *p* < 0.001, η_p_^2^ = 0.34, and fifth blocks, *F*(1,78) = 34.04, *p* < 0.001, η_p_^2^ = 0.30. Additionally, the difference between match and mismatch trials was approaching significance in Block 1, with higher accuracy on mismatching pairs, *F*(1,78) = 3.96, *p* = 0.05, η_p_^2^ = 0.05. There was also a simple main effect of block on match trials, *F*(4,75) = *p* < 0.001, η_p_^2^ = 0.63. Bonferroni-adjusted pairwise comparisons showed that this was due to higher accuracy in all blocks following the 10- and 8-second blocks, as well as in the 2-second block compared to the 6-second block, *p* < 0.01. No other comparisons were significant, all *p*s ≥ 0.17. Accuracy on mismatch trials deteriorated over the task, *F*(4,75) = 8.81, *p* < 0.001, η_p_^2^ = 0.32, with higher accuracy in Block 1 compared to all other blocks, all *p*s < 0.05, and in Block 2 compared to Block 4, *p* < 0.01. No other comparisons were significant, all *p*s ≥ 0.10.

*d-prime and criterion*

A 2 (time pressure) x 5 (time) mixed-factor ANOVA did not reveal an effect of time, *F*(4,312) = 2.04, *p* = 0.09, η_p_^2^ = 0.03, or of time pressure, *F*(1,78) = 0.74, *p* = 0.39, η_p_^2^ = 0.01, but a significant interaction, *F*(4,312) = 2.75, *p* < 0.05, η_p_^2^ = 0.03. Simple main effects analysis revealed that sensitivity was higher in Block 1 when time pressure was increasing (10 seconds), compared to when decreasing (2 seconds), *F*(1,78) = 7.76, *p* < 0.01, η_p_^2^ = 0.09. However, performance was comparable between the second, *F*(1,78) = 1.58, *p* = 0.21, η_p_^2^ = 0.02, third, *F*(1,78) = 0.08, *p* = 0.77, η_p_^2^ = 0.001, fourth, *F*(1,78) = 0.02, *p* = 0.90, η_p_^2^ = 0.00, and final block, *F*(1,78) = 1.89, *p* = 0.18, η_p_^2^ = 0.02. In addition, a simple main effect of time was found for the increasing time pressure condition, *F*(4,75) = 3.06, *p* < 0.05, η_p_^2^ = 0.14. Bonferroni-adjusted comparisons showed that this was due to lower *d’* in the 4-second block, compared to the 10-second block, *p* < 0.05. No other comparisons reached significance, all *p*s ≥ 0.06, and *d’* was comparable throughout the decreasing time pressure condition, *F*(4,75) = 1.09, *p* = 0.37, η_p_^2^ = 0.06.

Analysis of *criterion* did not reveal an effect of time pressure condition, *F*(1,78) = 1.05, *p* = 0.31, η_p_^2^ = 0.01, or a two-way interaction, *F*(4,312) = 0.45, *p* = 0.77, η_p_^2^ = 0.01. However, there was an effect of time, *F*(4,312) = 26.09, *p* < 0.001, η_p_^2^ = 0.25, due to a shift in response criterion over the task. Bonferroni-adjusted pairwise comparisons reflected that *criterion* was significantly higher in the 10-second block compared to all other blocks, all *p*s < 0.001, but also in the 8-second and 6-second block compared to the 4-second and 2-second blocks, both *p*s < 0.05. No further comparisons were significant, all *p*s ≥ 0.86.

These *criterion* scores were also compared to zero using one-sample *t*-tests. When time pressure was increasing, this showed that *criterion* was comparable to zero in the 10-second block, *t*(39) = 1.72, *p* = 0.09, but was below zero in the 8-second, *t*(39) = 2.14, *p* < 0.05, 6-second, *t*(39) = 2.66, *p* < 0.05, 4-second, *t*(39) = 5.23, *p* < 0.001, and 2-second block, *t*(39) = 5.30, *p* < 0.001. When time pressure was decreasing, *criterion* was comparable to zero in the 2-second, *t*(39) = 1.71, *p* = 0.09, and 4-second blocks, *t*(39) = -0.78, *p* = 0.44. However, *criterion* was reliably below zero in the 6-second, *t*(39) = 2.13, *p* < 0.05, 8-second, *t*(39) = 3.53, *p* < 0.01, and 10-second blocks, *t*(39) = 2.86, *p* < 0.01.

**
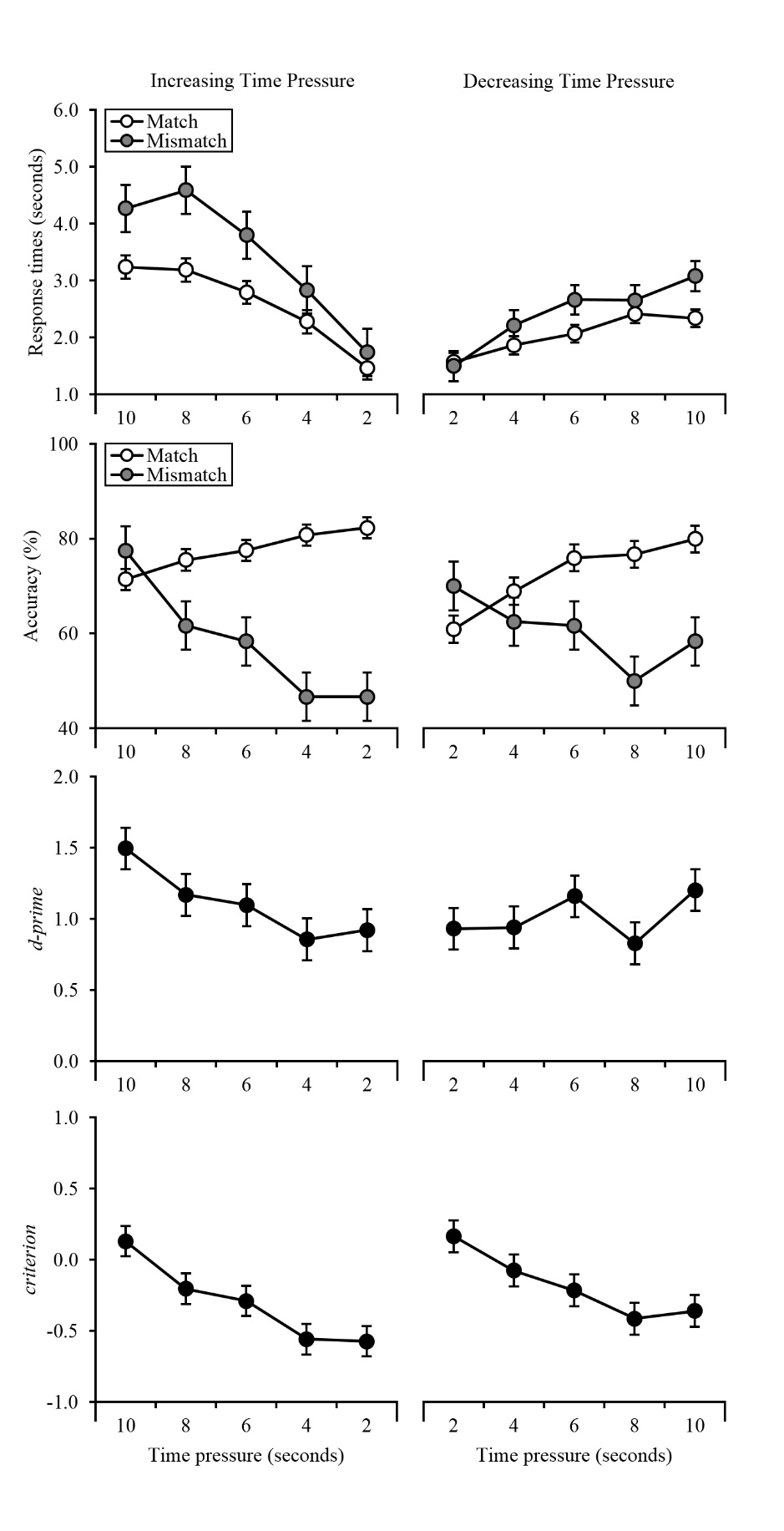
**

**Supplementary Material for Experiment 1.** Mean correct response times, percentage accuracy, d', and criterion across the increasing and decreasing time pressure conditions in Experiment 1. Open markers denote match trials, and grey markers denote mismatch trials. Error bars represent the standard error of the mean.

**Supplementary Data - Experiment 2**

*Response times*

A 2 (time pressure: increasing vs. decreasing) x 2 (trial: match vs. mismatch) x 4 (time: 8, 6, 4, 2) mixed-factor ANOVA revealed an effect of time pressure, *F*(1,51) = 12.02, *p* < 0.01, η_p_^2^ = 0.19, and of trial, *F*(1,51) = 16.84, *p* < 0.001, η_p_^2^ = 0.25. In addition, there was an effect of time, *F*(3,153) = 6.78, *p* < 0.001, η_p_^2^ = 0.12, which interacted with time pressure, *F*(3,153) = 55.54, *p* < 0.001, η_p_^2^ = 0.52. However, trial did not interact with time, *F*(3,153) = 2.42, *p* = 0.07, η_p_^2^ = 0.05, or with time pressure, *F*(1,51) = 0.91, *p* = 0.35, η_p_^2^ = 0.02, and the three-way interaction was not significant, *F*(3,153) = 0.74, *p* = 0.53, η_p_^2^ = 0.01.

For the interaction between time pressure and time, simple main effects analysis revealed that responses became faster as time pressure increased, *F*(3,49) = 42.89, *p* < 0.001, η_p_^2^ = 0.72, and pairwise comparisons showed that observers were quicker to respond in each subsequent block, all *p*s < 0.05. Conversely, responses became slower throughout the decreasing time pressure condition, *F*(3,49) = 7.80, *p* < 0.001, η_p_^2^ = 0.32. Pairwise comparisons revealed that this was due to slower responses in Blocks 3 and 4, compared to Blocks 1 and 2, all *p*s < 0.01. Response times were comparable between the 2-second and 4-second blocks, *p* = 0.50, and between the 6-second and 8-second block, *p* = 0.73. Comparing response speeds between conditions revealed that in the decreasing time pressure condition, responses were significantly faster in Block 1 (2 seconds), *F*(1,51) = 51.56, *p* < 0.001, η_p_^2^ = 0.50, and in Block 2 (4 seconds), *F*(1,51) = 38.77, *p* < 0.001, η_p_^2^ = 0.43, compared to Block 1 (8 seconds) and Block 2 (6 seconds) of the increasing time pressure condition. However, response speeds were comparable between conditions in the third block, *F*(1,51) = 0.55, *p* = 0.46, η_p_^2^ = 0.01, but were faster in Block 4 (2 seconds) when time pressure was increasing, compared to when decreasing (8 seconds), *F*(1,51) = 24.55, *p* < 0.001, η_p_^2^ = 0.33.

*Accuracy*

A 2 (time pressure) x 2 (trial) x 4 (time) mixed-factor ANOVA revealed a three-way interaction, *F*(3,174) = 5.09, *p* < 0.01, η_p_^2^ = 0.08. To interpret this, separate 2 (trial) x 4 (time) within-subjects ANOVAs were conducted for each time pressure condition.

For the increasing time pressure condition, this analysis did not reveal an effect of time, *F*(3,87) = 1.47, *p* = 0.23, η_p_^2^ = 0.05, but an effect of trial, *F*(1,29) = 6.34, *p* < 0.05, η_p_^2^ = 0.18, and a significant interaction, *F*(3,87) = 8.98, *p* < 0.001, η_p_^2^ = 0.24. Simple main effects analysis revealed that this was due to an improvement in accuracy on match trials across blocks, *F*(3,27) = 8.75, *p* < 0.001, η_p_^2^ = 0.49, with pairwise comparisons showing a significant improvement in performance from the 8-second condition, to the 4-second and 2-second condition, *p* < 0.01. In addition, an improvement was also observed between the 6-second and 2-second block, *p* < 0.05. Performance was comparable between the 8-second and 6-second blocks, and between the 6-second and 4-second blocks, both *p*s ≥0.10. Mismatch accuracy also deteriorated over the task, *F*(3,27) = 4.09, *p* < 0.05, η_p_^2^ = 0.31, due to worse performance in the 2-second condition compared to the 8-second condition, *p* < 0.05. No further comparisons reached significance, all *p*s ≥ 0.08. This analysis also revealed that accuracy was higher for match, compared to mismatch trials, in the 4-second block, *F*(1,29) = 8.97, *p* < 0.01, η_p_^2^ = 0.24, and the 2-second block, *F*(1,29) = 17.13, *p* < 0.001, η_p_^2^ = 0.37, compared to on mismatch trials. Performance between match and mismatch trials was comparable between the 8-second, *F*(1,29) = 0.28, *p* = 0.60, η_p_^2^ = 0.01, and 6-second blocks, *F*(1,29) = 0.14, *p* = 0.71, η_p_^2^ = 0.01.

For the decreasing time pressure condition, a 2 (trial) x 4 (time) within-subjects ANOVA did not reveal a significant interaction, *F*(3,87) = 1.23, *p* = 0.31, η_p_^2^ = 0.04, but an effect of trial, *F*(1,29) = 4.89, *p* < 0.05, η_p_^2^ = 0.14, due to higher accuracy on match, compared to mismatch trials. The effect of time was not significant, *F*(3,87) = 2.50, *p* = 0.07, η_p_^2^ = 0.08.

*d-prime and criterion*

For *d’*, a 2 (time pressure) x 4 (time) mixed-factor ANOVA did not reveal an effect of time pressure, *F*(1,58) = 0.08, *p* = 0.78, η_p_^2^ = 0.00, or of time, *F*(3,174) = 0.43, *p* = 0.73, η_p_^2^ = 0.01, but an interaction, *F*(3,174) = 3.87, *p* < 0.05, η_p_^2^ = 0.06. Simple main effects for this interaction showed that sensitivity improved under decreasing time pressure, *F*(3,56) = 3.98, *p* < 0.05, η_p_^2^ = 0.18. However, none of the pairwise comparisons between blocks reached significance, all *p*s ≥ 0.08. The deterioration in *d’* when time pressure was increasing was not significant, *F*(3,56) = 1.46, *p* = 0.24, η_p_^2^ = 0.07, and was comparable between increasing and decreasing time pressure conditions in the first, *F*(1,58) = 1.04, *p* = 0.31, η_p_^2^ = 0.02, second, *F*(1,58) = 3.62, *p* = 0.06, η_p_^2^ = 0.06, and third blocks, *F*(1,58) = 2.53, *p* = 0.12, η_p_^2^ = 0.04. The difference between the final block of the increasing time pressure condition (2 seconds) and the decreasing condition (8 seconds) was approaching significance, *F*(1,58) = 3.95, *p* = 0.05, η_p_^2^ = 0.06.

Analysis of *criterion* did not find an effect of time pressure, *F*(1,58) = 0.29, *p* = 0.60, η_p_^2^ = 0.01, but of time, *F*(3,174) = 6.14, *p* < 0.01, η_p_^2^ = 0.10, and a significant two-way interaction, *F*(3,174) = 4.42, *p* < 0.01, η_p_^2^ = 0.07. Analysis of simple main effects revealed that response criterion shifted under increasing time pressure, *F*(3,56) = 8.29, *p* < 0.001, η_p_^2^ = 0.31. Bonferroni-adjusted pairwise comparisons showed that *criterion* was significantly lower in in the 2-second block compared to the 6- and 8-second block, and in the 4-second block compared to the 8-second block, all *p*s < 0.05. No other comparisons were significant, all *p*s ≥ 0.06. Additionally, *criterion* was stable throughout the decreasing time pressure condition, *F*(3,56) = 1.53, *p* = 0.22, η_p_^2^ = 0.08, and was comparable between conditions in the first, *F*(1,58) = 2.46, *p* = 0.12, η_p_^2^ = 0.04, third, *F*(1,58) = 0.48, *p* = 0.49, η_p_^2^ = 0.01, and fourth block, *F*(1,58) = 0.41, *p* = 0.52, η_p_^2^ = 0.01, but was approaching significance in the second block, *F*(1,58) = 3.87, *p* = 0.05, η_p_^2^ = 0.06.

One-sample *t*-tests were conducted to compare *criterion* to zero in each block. In the increasing time pressure condition, *criterion* was reliably below zero in the 4-second *t*(29) = 2.58, *p* < 0.05, and 2-second blocks, *t*(29) = 3.90, *p* < 0.01, but not in the 8-second and 6-second blocks, both *t*s ≤ 0.81, both *p*s ≥ 0.42. For the decreasing time pressure condition, *criterion* was reliably below zero in Block 2 (4 seconds), *t*(29) = 2.52, *p* < 0.05, and Block 4 (8 seconds), *t*(29) = 2.08, *p* < 0.05, but was comparable to zero in Block 1 (2 seconds) and Block 3 (6 seconds), both *t*s ≤ 1.35, both *p*s ≥ 0.19.


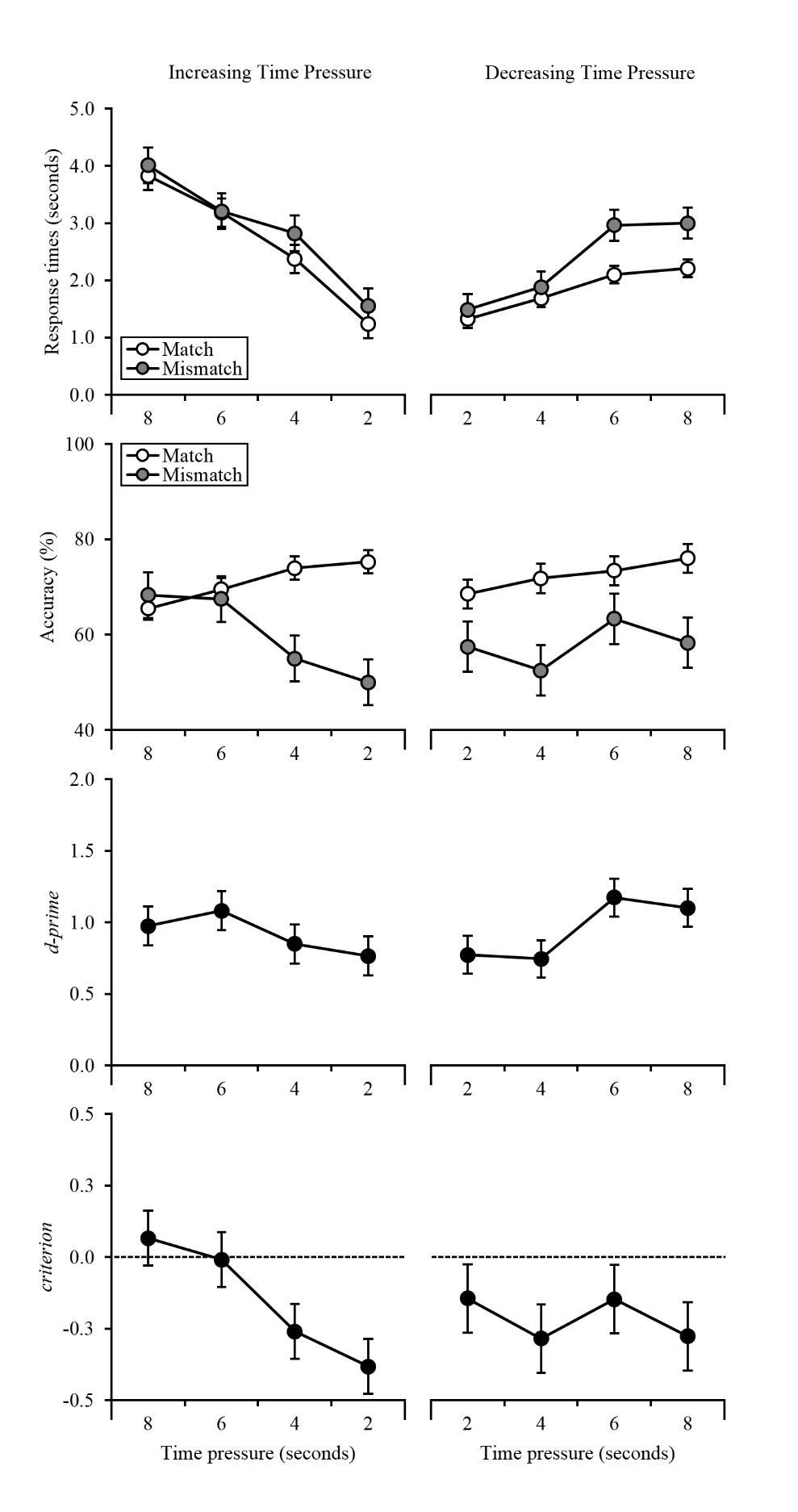


**Supplementary Material for Experiment 2.** Mean correct response times, percentage accuracy, d', and criterion across the increasing and decreasing time pressure conditions in Experiment 2. Open markers denote match trials, and grey markers denote mismatch trials. Error bars represent the standard error of the mean.
